# Fatal bacteremia due to immotile *Vibrio cholerae *serogroup O21 in Vientiane, Laos – a case report

**DOI:** 10.1186/1476-0711-7-10

**Published:** 2008-04-25

**Authors:** Rattanaphone Phetsouvanh, Masami Nakatsu, Eiji Arakawa, Viengmone Davong, Manivanh Vongsouvath, Olay Lattana, Catrin E Moore, Satoshi Nakamura, Paul N Newton

**Affiliations:** 1 Wellcome Trust-Mahosot Hospital-Oxford Tropical Medicine Research Collaboration, Microbiology Laboratory, Mahosot Hospital, Vientiane, Lao PD; 2 Department of Appropriate Technology Development and Transfer, Research Institute, International Medical Centre of Japan, Tokyo, Japan; 3 Department of Bacteriology, National Institute of Infectious Diseases, Tokyo, Japan; 4 Centre for Tropical Medicine, Nuffield Department of Clinical Medicine, Churchill Hospital, University of Oxford, Oxford, UK

## Abstract

**Background:**

Human infections with non-O1, non-O139 *V. cholerae *have been described from Laos. Elsewhere, non cholera-toxin producing, non-O1, non-O139 *V. cholerae *have been described from blood cultures and ascitic fluid, although they are exceedingly rare isolates.

**Case presentation:**

We describe a farmer who died with *Vibrio cholerae *O21 bacteremia and peritonitis in Vientiane, Laos, after eating partially cooked apple snails (*Pomacea canaliculata*) and mussels (*Ligumia *species). The cultured *V. cholerae *were non-motile. PCR detected *ompW *and *toxR *gene regions but not the *ctxA, ompU, omp K *and *TCP *gene regions. Although the organisms lacked flagellae on scanning electron microscopy, they possessed the *Vibrio *flagellin *flaA *gene.

**Conclusion:**

Severe bacteremic non-O1, non-O139 *V. cholerae *is reported from Laos. The organisms were unusual in being non-motile. They possessed the *Vibrio *flagellin *flaA *gene. Further research to determine the reasons for the non-motility and virulence is required.

## Background

Cholera is endemic in Laos and O1 and non-O1 *V. cholerae *(including serogroups 11, 14, 16, 21, 41, 43, 68 and 169), but not 0139, have been recorded [[1,2], Phouthavane *et al*. unpublished]. Non cholera-toxin producing, non-O1, non-O139 *V. cholerae *have been described from blood cultures and ascitic fluid, although they are exceedingly rare isolates. Consumption of undercooked shellfish, exposure of open wounds to salt and fresh water, drinking of well water, ascites, cirrhosis, renal failure and haematological malignancies have all been associated with non-O1, non-O139 disease [3-5]. The mortality of recorded non-O1 bacteraemia is high at 24–62% [6-11]. Concurrent infection with organism(s) that may damage the bowel mucosa may predispose patients to *V. cholerae *non-O1 bacteraemia [12]. Of 28 patients with non-01 *V. cholerae *bacteremia recorded in Taiwan over 5 years, 95% had ascites and 75% had cirrhosis, perhaps because increased intestinal permeability in cirrhotic patients predisposes them to invasive disease [13]. Unlike O1 biotype El Tor, but like O139 *V. cholerae*, O21 possess a capsule, which is thought to facilitate survival in the blood stream [12]. *V. cholerae *01 bacteremia has also been described, but is more rare than non-01 [14].

## Case Presentation

In January 2006, a 20 year old female rice farmer, from Nayxaythong District, on the outskirts of Vientiane City, was admitted to Mahosot Hospital, Vientiane, Lao PDR (Laos) with a 7 day history of abdominal pain, fever, rigors, watery diarrhoea, yellow eyes, leg oedema, anuria and malaena. She had collected many apple snails 'hoy pak kwang' (*Pomacea canaliculata) *and mussels 'hoy kee' (*Ligumia *species) from the mud on the edge of a nearby lake and washed them before boiling them for 30 minutes. She ate partially cooked snails as she boiled them and became unwell 7 days later. The fully cooked snails were eaten, with chilli sauce, by another villager who remained well. On admission she was had a fever (39.5°C) with jaundice, abdominal tenderness, splenomegaly, tachycardia, shortness of breath, lower leg oedema and ascites. She had no skin lesions and had no evidence for prior cirrhosis, antacid or steroid use, gastric surgery or haemoglobinopathy. Her haematocrit was 35%, with a peripheral white cell count of 8.9 10^9^/L (66% neutrophils). Serum creatinine, alkaline phosphatase and glucose were within the reference ranges, with raised total serum bilirubin 41 μmol/L (reference <14.5 μmol/L), AST 75 IU/L (<37 IU/L) and ALT 170 IU/L (<40 IU/L). Abdominal ultrasound demonstrated ascites and splenomegaly with normal liver and kidneys. Stool was not cultured. She was treated empirically with intravenous ampicillin, gentamicin and diuretics. Analysis of tapped ascitic fluid revealed many white cells, 90% of which were neutrophils.

Oxidase positive, curved, non-motile Gram negative rods were grown from 2 of 2 blood cultures, after 2 days incubation and the ascitic fluid, after 1 day of incubation. API 20E (bioMeriuex, France) identified *Vibrio cholerae *with 99.9% confidence. The organism grew in 0% sodium chloride but not in 6% or 10% sodium chloride and did not agglutinate when tested against O1 and O139 antisera, but did against O21 antisera (Department of Bacteriology, National Institute of Infectious Disease in Tokyo, Japan). By disc diffusion testing (Kerby-Bauer, according to NCCLS guidelines 2003) the organisms were susceptible to ampicillin, ciprofloxacin, ceftriaxone and tetracycline. She was taken home moribund, at her relatives' request, after 2 days in hospital and died soon afterwards.

In order to investigate whether we could grow 021 *V. cholerae *from the probable source of infection, in May 2006 specimens of *P. canaliculata *and *Ligumi *species were collected from the same lake as above and cultured in TCBS according to the methods of Ottaviani *et al*. [15]. No *V. cholerae *were grown but *Aeromonas, Enterobacter, Proteus *and *Escherichia *species were identified.

**Figure 1:**
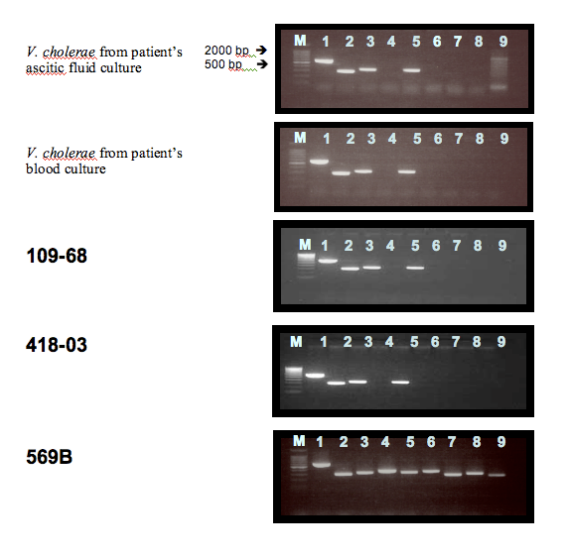
**Agarose gel electrophoresis analysis of *V. cholerae *from patient's ascitic fluid culture and blood culture isolates, 109-68, 418-02 and 569B**. Lane M is the 100 bp lambda ladder marker (Toyobo, Japan); lanes 1–4 are ompW (the primer pairs 1/2, 1/4, 2/4, F/R) and lanes 5–9 are *toxR, ctxA, ompU, ompK *and *TCP*.

**Figure 2:**
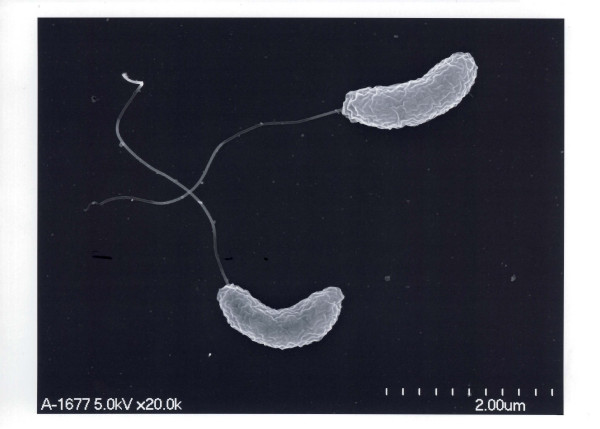
Scanning electron micrograph of an environmental *V. cholerae *isolate from USA (418-03).

**Figure 3:**
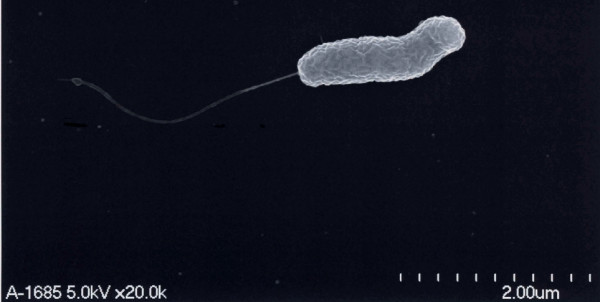
Scanning electron micrograph of a human *V. cholerae *isolate from India (109-68).

**Figure 4:**
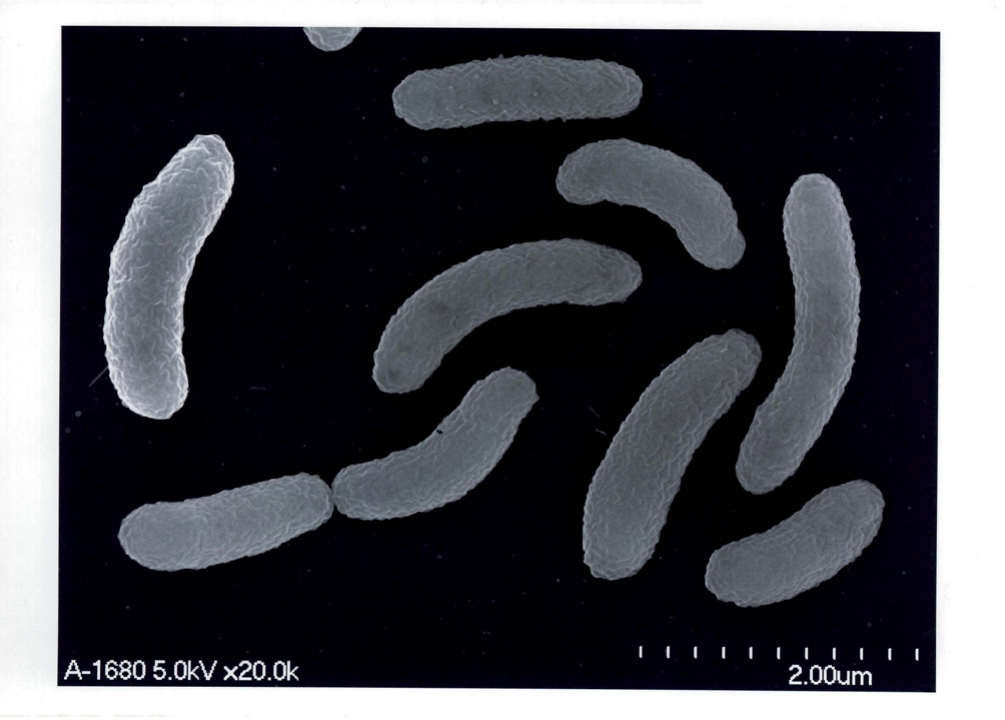
Scanning electron micrograph of *V. cholerae *organisms cultured from the patient, demonstrating the absence of flagellae.

The PCR techniques of Nandi *et al*. [16] were used to confirm that the organisms contained the outer membrane protein W (ompW) and regulatory protein (toxR) of *Vibrio cholerae *and to determine whether they contained cholera toxin (ctxA) and toxin-coregulated pilus (TCP) virulence genes (Table 1 &2). The bacteria from blood culture and ascitic fluid were re-plated on Modified Drigalski Agar (BTB agar, Eiken Kagaku Co Ltd, Tokyo, Japan) to obtain pure growth and DNA extraction was performed (Wizard, A1120, Promega, WI, USA). Biotype Classic O1 Inaba strain 569B and *Vibrio cholerae *O21, recovered from a patient with diarrhoea in Vientiane Capital, were used as positive control and reference strains, respectively and compared with human isolates of serogroup O21 109-68 from India (from diarrhoeal stool), and environmental isolate 418-03 from the USA (seawater, Chesapeake Bay, Virginia) (both from National Institute of Infectious Diseases (NIID) Tokyo). Primers of Outer membrane protein W (*ompW*) were prepared according to [16] and outer membrane protein U (*ompU*), outer membrane protein K (*ompK*), toxin-coregulated pilus (TCP), cholerae toxin A (*ctx*A) and Regulatory protein (*tox*R) primers were designed (Table 2) and DNA amplification performed (i-cycler, BioRad) with analysis using 1.8% agarose gel electrophoresis. *V. cholerae *O21 cultures from both blood and ascitic fluid from our patient and 109-68 and 418-03 had *ompW *and *toxR *gene regions, confirming them to be *V. cholerae *[16], but had no *ctxA, ompU, omp K, TCP *gene regions, which were present in the Classic 569B strain (Figure 1, Table 1). Scanning electron microscopy (SEM; S-5200, Hitachi, Japan) of the patient's isolate, serogroup O21 109-68 and 418-03 (Figures 2, 3, 4) demonstrated that, although 109-68 and 418-03 did possess flagellae, the organisms isolated from the patient did not. The flagellin regulation gene flaA was demonstrated by PCR in the patient's isolates and 569B with specific oligonucleotide primers (Table 3), which we designed based on the work of Klose and Mekalanos [17] who demonstrated that immotility could be caused by mutations in the *flaA *gene alone.

**Table 1:** Summary of the gene regions detected in the patients isolates from ascitic fluid and blood culture compared to the 569B reference organism and serogroup O21 609-68 and 418-03 strains kindly provided by the National Institute of Infectious Diseases, Tokyo, Japan.

Gene	Primer	Product length/bp	Inaba 569B	609-68 India	418-03 USA	Patient's ascitic fluid	Patient's blood culture
*ompW*	ompW 1/2	588	+	+	+	+	+
*ompW*	ompW1/4	304	+	+	+	+	+
*ompW*	ompW 2/4	336	+	+	+	+	+
*ompW*	ompW F/R	373	+	-	-	-	-
*ToxR*	Tox F/R	337	+	+	+	+	+
*ctxA*	ctxA F/R	354	+	-	-	-	-
*ompU*	ompU F/R	283	+	-	-	-	-
*ompK*	ompK F/R	310	+	-	-	-	-
*TCP*	TCP F/R	265	+	-	-	-	-

Notes. Amplification by was done in a 25 μl reaction mixture containing 3 μl of template DNA, 200 μM of each dNTP, 1X reaction buffer, 1.25 units of Taq polymerase (TaKaRa Ex-Taq), and 0.5 μM of each primers. The initial amplification conditions were one cycle at 95°C for 7 minutes, 35 cycles at 95°C for 30 seconds. 55°C for 30 seconds, and 72°C for 30 seconds; and elongation step of 72°C for 5 minutes. Nested PCR was performed with the same method except that the annealing temperature was 60°C and the template was 2 μl of the first PCR product. Electrophoresis was performed on a 1.8% agarose gel.

**Table 2:** Primers and primer sequences. From reference [16] and this paper

Primer name	Sequence	Accession number if designed in this experiment
ompW-1	CACCAAGAAGGTGACTTTATTGTG	[16]
ompW-2	GAACTTATAACCACCCGCG	[16]
ompW-3	CCACCTACCTTTATGGTCC	[16]
ompW-4	GGAAAGTCGAATTAGCTTCACC	[16]
ompW-F	GTTTTTGAAGTCCTCGCTGCT	NC_002506
ompW-R	GCATCTGCACCTGCTTTGTA	NC_002506
ctxA-F	TCAGACGGGATTTGTTAGGC	AF463400
ctxA-R	CCTGCCAATCCATAACCATC	AF463400
toxR-F	GATTAGGCAGCAACGAAAGC	M21249
toxR-R	GATGAAGGCACACTGCTTGA	M21249
ompU-F	GCTGTAGCAGACCGTGTTGA	NC_002505
ompU-R	GGTTTTCCATGCGGTAAGAA	NC_002505
ompK-F	GCAACGAACAAAAGCAGTGA	NC_002505
ompK-R	ACCAGTTGGTCGAGATTTGG	NC_002505
TCP-F	TGGGCAGATATTTGTGGTGA	NC_002505
TCP-R	TTTCTGCAACTCCTGTCAACAT	NC_002505

**Table 3:** Primers used for PCR of the flaA gene of *V. cholerae *(designed using *V. cholerae *accession number AF007121 and the JustBio primer soft software)

Primer name	Sequence	Product length (bp)
FlaA_F1	GACCGCACAACGTTATCTGA	321
FlaA_R1	AGTGCCACCGACTCTTCATT	
FlaA_F2	AACCGTATCGCTGAAACCAC	323
FlaA_R2	TCCGTTTGACCGTTGATGTA	
FlaA_F3	AAGCTTCGGTTGACCAAGAA	338
FlaA_R3	TCCTTCGCAAAATCCGTATC	

The 021 *V. cholerae *cultured from blood and ascitic fluid of our patient were non-motile. *V. cholerae *are normally motile, although non-motile variants of Classical O1 may arise in the laboratory [17,18] and non-01 non-motile clinical isolates have been described [19]. The combination of SEM and PCR demonstrated that the organisms lacked flagellae but did possess the important regulatory flagellin gene *flaA *on the genome. The underlying reasons for the absence of flagellae in the patient's isolates are being investigated.

Non-01 *V. cholerae *bacteremia has been described from Thailand [9,20] and recently from Laos (Phouthavane *et al*. unpublished). Our patient may have contracted the infection from the molluscs or from injuries whilst walking barefoot on the edge of the lake. An association between *V. cholerae *and non-biting chironomid midges (Diptera; Chironomidae) egg masses, which live in fresh water, has been described [21] and such midges may be important in the ecology of cholera. The collection and consumption of snails are an important part of life in Laos, as in rural communities in Australia and USA where non O1 *V. cholerae *bacteremia has been described amongst people who fish [22]. *V. cholerae *are usually associated with water of medium salinity as it requires trace amounts of sodium chloride for growth but it can be contracted from fresh water environments [4,8]. Although non-01 and non-0139 *V. cholerae *are not usually associated with epidemics, in Bangladesh non-01 *V. cholerae *has caused outbreaks [23]. The optimum treatment remains uncertain but third generation cephalosporins, doxycycline and fluoroquinolones have been suggested [3].

## Conclusion

Fatal bacteremic non-O1, non-O139 *V. cholerae *is reported from Laos. The organisms were unusual in being non-motile. They did however possess the *Vibrio *flagellin *flaA *gene. Further research on the determinants of the non-motility is required. Given the importance of fishing and water in Lao society and the high prevalence of cirrhosis secondary to hepatitis B and C infection [24], *V. cholerae *non-01 bacteraemia may be a more important disease than this case report suggests.

## Competing interests

The authors declare that they have no competing interests.

## Authors' contributions

RP was in charge of diagnosis and edited the ms, MN performed PCR assays and helped write ms, EA performed diagnosis work up and SEM, and edited ms, VD, MV and OL performed diagnosis work up and edited ms, CEM assisted with diagnostic workup, reviewed molecular analysis and edited ms, SN performed diagnosis workup and helped write ms and PNN assisted with diagnostic workup, wrote the first draft and edited ms. All authors read and approved the final manuscript.
